# Supplementary material for: Advancing Extrapulmonary Tuberculosis Diagnosis: Potential of MPT64 Immunochemistry-Based Antigen Detection Test in a High-TB, Low-HIV Endemic Setting
Source: Pathogens. 2025 Jul 28;14(8):741. doi: 10.3390/pathogens14080741 (PMC12388923; doi:10.3390/pathogens14080741)
Supplement: Supplementary file 1 [file pathogens-14-00741-s001.zip › pathogens-3745075-supplementary.pdf]

Table S1. Comparison of antigen load detected by the MPT64 test with respect to clinical features of patients.

**Clinical features**	**n**	**Mild-moderate**	**Strong**
		**n (%)**	**n (%)**
Culture positive	113	80 (71%)	33 (29)
Xpert MTB detected	82	60 (73%)	22 (27)
HPG-1	3	3 (100%)	0 (0%)
HPG-2	68	42 (62%)	26 (38%)
HPG-3	51	37 (73%)	14 (27%)
HPG-4	10	8 (80%)	2 (20%)
Delayed	121	89 (74%)	32 (26%)
Not delayed	98	61 (62%)	37 (38)
TBLN			
Severe	45	31 (69%)	14 (31%)
Not severe	108	71 (66%)	37 (34%)
TBP			
Severe	21	13 (62%)	8 (38%)
Not severe	47	35 (74%)	12 (26%)

HPG, histopathological groups; MTB, *Mycobacterium tuberculosis*; TBLN, tuberculous lymphadenitis; TBP, tuberculous pleuritis.

HPG-1: well-formed granulomas without necrosis; HPG-2: well-formed granulomas with necrosis; HPG-3: ill-formed granulomas with necrosis; HPG-4: necrosis only.

- Delayed refers to the period from symptom onset to enrollment for anti-tuberculosis treatment.
- Severity is based on clinical symptoms, cytology, and radiological findings.
